# Secretome Profiling of Periodontal Ligament from Deciduous and Permanent Teeth Reveals a Distinct Expression Pattern of Laminin Chains

**DOI:** 10.1371/journal.pone.0154957

**Published:** 2016-05-05

**Authors:** Priscila A. Giovani, Cristiane R. Salmon, Luciane Martins, Adriana F. Paes Leme, Pedro Rebouças, Regina M. Puppin Rontani, Luciana S. Mofatto, Enilson A. Sallum, Francisco H. Nociti, Kamila R. Kantovitz

**Affiliations:** 1 Department of Pediatric Dentistry, Piracicaba Dental School, University of Campinas, São Paulo, Brazil; 2 Department of Prosthodontics and Periodontics, Division of Periodontics, Piracicaba Dental School, University of Campinas, São Paulo, Brazil; 3 Brazilian Biosciences National Laboratory, LNBio, CNPEM, Campinas, São Paulo, Brazil; 4 Department of Genetics, Institute of Biology, University of Campinas, Campinas, São Paulo, Brazil; 5 Department of Dental Materials, São Leopoldo Mandic Research Center, Campinas, São Paulo, Brazil; University of Bergen, NORWAY

## Abstract

It has been suggested that there are histological and functional distinctions between the periodontal ligament (PDL) of deciduous (DecPDL) and permanent (PermPDL) teeth. Thus, we hypothesized that DecPDL and PermPDL display differences in the constitutive expression of genes/proteins involved with PDL homeostasis. Primary PDL cell cultures were obtained for DecPDL (n = 3) and PermPDL (n = 3) to allow us to perform label-free quantitative secretome analysis. Although a highly similar profile was found between DecPDL and PermPDL cells, comparative secretome analysis evidenced that one of the most stickling differences involved cell adhesion molecules, including laminin subunit gamma 1 (LAMC1) and beta 2 (LAMB2). Next, total RNA and protein extracts were obtained from fresh PDL tissues of deciduous (n = 6) and permanent (n = 6) teeth, and Western blotting and qPCR analysis were used to validate our *in vitro* findings. Western blot analysis confirmed that LAMC1 was increased in DecPDL fresh tissues (p<0.05). Furthermore, qPCR data analysis revealed that mRNA levels for laminin subunit beta 1 (*LAMB1)*, beta 3 (*LAMB3)*, *LAMC1*, and gamma 2 (*LAMC2)* were higher in DecPDL fresh tissues, whereas transcripts for *LAMB2* were increased in PermPDL (p<0.05). In conclusion, the differential expression of laminin chains in DecPDL and PermPDL suggests an involvement of laminin-dependent pathways in the control of physiological differences between them.

## Introduction

The periodontal ligament (PDL) has been shown to be a highly specialized connective tissue that connects the tooth root to the adjacent alveolar bone [1]. The PDL tissue is characterized by several cell types, including fibroblasts, progenitor cells, epithelial cell rests of Malassez, cementoblasts, vascular cells, nerve cells, osteoblasts, and osteoclasts [2]. In health, the PDL functions have been reported to involve tooth anchorage, nutrition, homeostasis, provision of proprioceptive information and repair of damaged tissues [3].

In humans, there are two distinct sets of dentitions, primary/deciduous and permanent, and a number of functional, anatomical and structural differences have been described between these two dentitions [4–7]. For instance, primary and permanent teeth exhibit distinct responses to external stimuli, and deciduous teeth present a less organized sensory system [8,9]. Clinically, the most evident difference between primary and secondary dentitions is the physiologic loss of the primary tooth by root resorption. Non-physiologic root resorption can occur in both dentition sets, however, as a consequence of orthodontic tooth movement, traumatic occlusion, pulpal inflammation and/or genetic reasons [10]. Based on the fact that deciduous teeth will eventually undergo physiologic root resorption, distinctions between PDL tissues from deciduous and permanent teeth have been addressed by few studies at the molecular level. Extracellular matrix degrading enzymes, including metalloproteinases and collagenase were found to be increased in the PDL tissues obtained from deciduous teeth under root resorption [11–13]. In addition, it was shown that PDL cells harvested from resorbing areas in deciduous teeth may present higher transcript levels for the receptor activator of nuclear factor-kappa B ligand (RANKL) [14,15], suggesting that, as reported for bone resorption, the RANK-RANKL-OPG system may play a key role in controlling the physiologic deciduous teeth root resorption. Furthermore, PDL tissues from deciduous teeth have been reported to express higher levels of osteopontin (OPN) and bone sialoprotein (BSP), which may favor odontoclasts binding [16,17]. Song et al. (2013) examined the expression of a large number of genes in the PDL tissues harvested from deciduous and permanent teeth using a transcriptomic approach [18]. A highly similar expression pattern was observed, and it was concluded that the differential expression of genes regulating the formation of extracellular matrix and inflammation/immune reactions in deciduous teeth might give support to the distinct functions of PDL tissues around deciduous and permanent teeth. Although, a significant progress has been made in recent years towards the better understanding of the dissimilarities between the PDL tissues from deciduous and permanent teeth, further work is required in order to explain the differences in the physiologic-functioning periodontium of these two distinct dentitions.

Proteomic-based techniques have been used in the field of biomedical sciences, and have produced a substantial amount of data especially regarding pathophysiological conditions leading to abnormal protein expression and dysfunctional phenotypes [19,20]. In dentistry, it is found the use of different proteomic approaches in a wide variety of organic samples, such as saliva, microorganisms, and different tissues (including normal or pathologic enamel, dentin, pulp, gingiva, bone, ligament, cementum, and mucosa, among others). In the current study, we took a comprehensive proteomic approach in order to generate a comparative secretome list for periodontal ligament cells harvested from permanent and deciduous teeth, and our initial findings were expanded and validated in fresh PDL tissues harvested from deciduous and permanent teeth using qPCR and Western blotting analysis. In contrast to previous studies, the present investigation tested the hypothesis that there are differential basal protein expression between PDL cells harvested from clinically healthy deciduous and permanent teeth, and it is expected that the data presented here can aid in clarifying distinct molecular functions played by the periodontium in its physiological state.

## Material and Methods

### 2.1 Study population

Completely erupted/root-formed permanent teeth (n = 3, 2 male and 1 female, aged 19 to 23 years) and deciduous teeth (n = 3, 2 male and 1 female, aged 8 to 10 years), with no signs of root resorption, were collected to establish primary PDL cell cultures. In addition, PDL tissues were obtained from healthy permanent (n = 6, 1 male and 5 female, aged 18 to 35 years) and deciduous teeth (n = 6, 3 male and 3 female, aged 5 to 8 years) for RNA and protein isolation. Teeth extractions were performed due to orthodontic (permanent teeth) and space management (deciduous teeth) reasons at the Dental Clinic of the School of Dentistry, University of Campinas, Piracicaba, São Paulo, Brazil, following a protocol approved by the Piracicaba Dental School—University of Campinas Institutional Review Board (#120/2014), and a written informed consent to participate of the study was obtained from all of the subjects and guardians of the children enrolled in this study, in compliance with the World Medical Association Declaration of Helsinki, Ethical Principles for Medical Research Involving Human Subjects, and the study was performed from 2014 to 2015.

### 2.2 Cell culture

Collected teeth were immediately placed in biopsy media composed of Dulbecco’s modified Eagle’s medium (DMEM) supplemented with 10% fetal bovine serum, 250 mg/mL gentamicin sulfate, 5mg/mL amphotericin B, and 1% penicillin/streptomycin (Gibco BRL, Rockville, MD, USA), and transferred to the laboratory facilities. The PDL tissues from deciduous (DecPDL) and permanent (PermPDL) teeth were gently scraped from the mid portion of the root surface and enzymatically digested in a solution of 3 mg/ml collagenase type I and 4 mg/mL dispase for 1 h at 37°C. Heterogeneous single-cell suspensions were obtained by passing the cells through a 70-mm cell strainer. Samples were expanded in 25 cm^2^ culture flasks at 37°C and 5% CO^2^ in DMEM supplemented with 10% fetal bovine serum, 1% L glutamine and 1% penicillin/ streptomycin (standard media), frozen in dimethyl sulfoxide, and kept in liquid nitrogen for subsequent experiments. For each experiment, cells in passages two to four were used, and experiments were performed simultaneously for both groups with cells within the same passage.

### 2.3 Functional characterization of collected cells

In order to determine whether the collected PermPDL and DecPDL cell populations presented the expected biological properties, PermPDL and DecPDL cells were seeded (6x10^4^ cells/cm^2^) in 24-well plates to assess their capacity to form mineral nodules *in vitro*. After 24 hs, standard medium was changed to an osteogenic-inducing medium (DMEM 10% FBS, 50 mg/mL ascorbic acid, 10 mM β-glycerol- phosphate, 10^−5^ M dexamethasone). Osteogenic medium was changed every other day and *in vitro* mineral nodule formation assessed after day 21 using the von Kossa assay.

### 2.4 Sample preparation

In order to collect culture media from PermPDL and DecPDL cells to differentially determine their secretome profile, cells were seeded at 8x10^5^ cells/plate in 100 mm culture dishes in standard medium until their were 70%-80% confluent. Standard medium was then changed to standard medium with no FBS for 24 h. Medium was collected and added with 1 mM EDTA and 1 mM PMSF. Samples were centrifuged at 4,000 x g during 5 minutes at 4°C to eliminate debris and concentrated by using a 3000-Da centrifugal filter (Sartorius Stedim Biotech GmbH, Goettingen, Niedersachsen, Germany) following manufacturer’s instructions. Proteins were denatured in 8M urea for 30 minutes at room temperature. Extracted proteins were reduced by incubation with 5 mM dithiothreitol at 56°C for 25 minutes and alkylated by incubation with 14 mM iodoacetamide at room temperature for 30 minutes in the dark, and further incubation in 5 mM dithiothreitol for 15 minutes at room temperature. Samples were then diluted into 50 mM ammonium bicarbonate to a final concentration of 1.6 M urea and 0.1 M calcium chloride was added to the mixture of proteins. Samples were digested by incubation with 2 ug of sequencing-grade modified trypsin (Promega, Madison, WI, USA) overnight at 37°C. After digestion peptide mixtures were equilibrated to pH 2.0 by addition of 0.4% trifluoroacetic acid. The tryptic peptides samples were desalted using Sep-pack cartridges (Waters Corporation, Milford, MA, USA) dried in a vacuum concentrator and then reconstituted in 0.1%formic acid, centrifuged at 10,000 rpm for 10 minutes and stored at -20°C for analysis.

### 2.5 Liquid chromatography-high resolution mass spectrometry (LC-MS/MS) analysis

An aliquot of 4.5 uL of the resulting mixture of peptides of each sample was loaded on a mass spectrometer LTQ Orbitrap Velos (Thermo Fisher Scientific, Waltham, MA, USA) connected to a nanoflow LC (LC-MS/MS) instrument by an EASY-NLC system (Proxeon Biosystems, West Palm Beach, FL, USA) with a Proxeon nanoelectrospray ion source. Peptides were separated by 2%-90% acetonitrile gradient in 0.1% formic acid using a PicoFrit analytical column (20 cm x ID75 μm, 5 μm particle size, New Objective, Woburn, MA, USA), with a flow rate of 300 nL/min for 45 minutes. The nanoelectrospray voltage was set to 1.7 kV and the source temperature was 275°C. All the equipment instrumental methods were set up in the data-dependent acquisition mode. Full scan of MS spectra (m/z 300–1600) were acquired by the Orbitrap analyzer after accumulation to a target value of 1e6. The resolution was set to the r = 60,000 and the 5 most intense peptide ions with charge states ≥ 2 were sequentially isolated to a target value of 5,000 and fragmented in the linear ion trap using low-energy CID (normalized collision energy of 35%). The signal threshold to trigger an MS/MS event was set to 500 counts. Dynamic exclusion was enabled with an exclusion size list of 500, exclusion duration of 60 seconds, and repeat count of 1. An activation q = 0.25 and activation time of 10 ms were used [21].

### 2.6 Data analysis

The MS/MS spectra (msf) were generated from the raw data files using Proteome Discover version 1.3 (Thermo Fisher Scientific) with Sequest (Thermo Finnigan, San Jose, CA, USA; version 1.4.0.288) search engine and searched against HUMAN_release22_01_2014.fasta (unknown version, 88429 entries) assuming a non-specific enzymatic digestion and with carbamidomethylation in cysteine residues (+57.021Da) as fixed modification, oxidation of methionine (+15,995 Da) as a variable modification, a tolerance of 10 ppm for precursor and 1.0 Da for fragment ions. For protein quantification, the data files were analyzed in Scaffold Q+ (version Scaffold_4.4.1.1, Proteome Software, Inc., Portland, OR, USA) and the quantitative value (normalized spectral counts) was obtained with the protein thresholds set at a minimum 90% probability and at least one peptide with thresholds established at a minimum 60% probability and XCorr cutoffs +1 > 1.8, +2 >2.2, +3 >2.5 and +4 >3.5 to have less than 1% FDR. Only peptides with a minimum of five amino acid residues, which showed significant threshold (p < 0.05) in the Sequest-based score system, were considered as a product of peptide cleavage. The peptide was considered unique when differed in at least 1 amino acid residue; covalently modified peptides, including N- or C-terminal elongation (i.e. missed cleavages) were counted as unique, and different charge states of the same peptide and modifications were not counted as unique.

### 2.7 PDL tissue preparation and gene/protein expression analysis

PDL samples were obtained from healthy functional permanent (n = 6) and deciduous (n = 6) for RNA and protein isolation from the same samples using the Trizol^®^ reagent method (Gibco BRL, Gaithersburg, MD, USA). The PDL tissues were carefully obtained immediately after extractions using sterile curettes from the middle-third of the tooth root and submerged in Trizol^®^. Next, PDL tissues were homogenized and total RNA and protein extracts obtained as recommended by the manufacturer. For gene expression analysis, cDNA was synthesized from 1 μg of total RNA using the Transcriptor first-strand cDNA synthesis kit (Roche Diagnostic Co., Indianapolis, IN, USA), and real-time quantitative PCR (qPCR) was performed by the Roche LightCycler 480 II system, with primers for *GAPDH* (5’-GAAGGTGAAGGTCGGAGTC-3’/5’-GAAGATGGTGATGGGATTTC-3’), *LAMA1* (laminin subunit alpha 1) (5’-AGGATGACCTCCATTCTGACTT-3’/5’-CCTTACATGGGCACTGACCT-3’), *LAMA4* (laminin subunit alpha 4) (5’-GACCCTGAGGACACAGTGTTTTA-3’/5’-AGGCAGGTTTAAGCTGGTAGG-3’), *LAMA5* (laminin subunit alpha 5) (5’-CCTCGTCCTCCAATGACAC-3’/5’-GCGCTGCAGTCACAATTC-3’), *LAMB1* (laminin subunit beta 1) (5’-AGTGCATGCCTGGGTTTG-3’/5’-CCTGGGGTCACAGTCACAG-3’), *LAMB2* (laminin subunit beta 2) (5’-CTGGTGGCAGTCAGAGAATG-3’/5’-CAGCAGGGCGAAATGTCT-3’), *LAMB3* (laminin subunit beta 3) (5’-AAAGAACGGCAGAACACACA-3’/5’-AGGGCAAAACACAAGAGGAA-3’), *LAMC1* (laminin subunit gamma 1) (5’-CTGTTACTAGCCTCCTCAGCATTA-3’/5’-GCTTATTCAGGTCCACTGTATCC-3’), and *LAMC2* (laminin subunit gamma 2) (5’-CAGAAGCCCAGAAGGTTGAT-3’/5’-ACACTGAGAGGCTGGTCCAT-3’). Western blotting analysis was used to determine LAMC1 protein levels in the PDL tissues harvested from permanent and deciduous teeth. After protein separation in 10% SDS-PAGE gel, proteins were electro-transferred onto nitrocellulose membranes. The membranes were incubated in blocking solution (5% non-fat skim milk in PBS containing 0.05% Tween-20) for 1 h at room temperature, then incubated with rabbit monoclonal anti-LAMC1 antibody (1:1,000, ab134059, Abcam^®^, Cambridge, MA, USA) overnight at 4°C. After washing and blocking, the membranes were further incubated with corresponding horseradish peroxidase-conjugated secondary antibody (1:20,000) for 1 h at room temperature. Membranes were washed, and the proteins recognized by the antibodies were visualized with chemiluminescent substrate (SuperSignal West Femto, Thermo Scientific) according to the manufacturer’s directions. Table 1 presents a detailed description of the evaluated laminin subunits.

**Table 1:** Laminin subunits assessed in the study.

Gene Symbol	Full Name	Chromosome	NCBI accession number		UniProtKB Swiss-Prot
			mRNA	Protein	
*LAMC1* *	Laminin subunit gamma 1 *	1q31	NM_002293.3	NP_002284.3	P11047
*LAMC2* *	Laminin subunit gamma 2 *	1q25-q31	NM_005562.2	NP_005553.2	Q13753
*LAMB1* *	Laminin subunit beta 1 *	7q22	NM_002291.2	NP_002282.2	P07942
*LAMB2* *	Laminin subunit beta 2 *	3q21	NM_002292.3	NP_002283.3	P55268
*LAMB3* *	Laminin subunit beta 3 *	1q32	NM_000228.2	NP_000219.2	Q13751
*LAMA1* *	Laminin subunit alpha 1*	18p11.3	NM_005559.3	NP_005550.2	P25391
*LAMA4* *	Laminin subunit alpha 4 *	6q21	NM_001105206.2	NP_001098676.2	Q16363
*LAMA5* *	Laminin subunit alpha 5 *	20q13.2-q13.3	NM_005560.4	NP_005551.3	O15230

* provided Hugo Gene Nomenclature Committee (HGNC; http://www.genenames.org/)

### 2.8 Histological analysis

In order to comparatively evaluate potential histological dissimilarities between the PDL tissues in DecPDL and PermPDL we used health BR1 male minipigs. Animals were kept in stalls at the University of Campinas—Dental School animal facility and fed with swine chow and wheat bran twice a day, and water *ad libitum*. Five months old (n = 3) and 18 months old (n = 3) animals were used to obtain mandible blocks with deciduous and permanent teeth, respectively. At sacrifice, the animals were anesthetized and perfused with a 10% formalin solution. Mandibles were dissected, trimmed, decalcified in Morse’s solution (50% formic acid, 20% sodium citrate), and paraffin embedded. Serial 6 μm thick sections were obtained in a mesio-distal direction and stained with Mallory’s trichrome and picrosirius red staining for bright field and polarizing microscope analysis, respectively. The experimental procedures were approved by the University of Campinas—Institutional Animal Care and Use Committee (protocol # 3129–1).

### 2.9 Statistical and differential analysis

For label-free quantification of endogenous peptides, the spectral count and the number of unique peptides were assessed. Resulting spectrum count values were used to analyze the abundance of identified proteins throughout the samples. Global normalization was applied to these counts and fold-changes were calculated using the mean of the normalized counts. Differential analysis was performed using beta-binomial test R package [22] on normalized spectral counts and *p* value threshold of < 0.05. The beta-binomial test is able to make a statistical model for the variation within the sample and between samples [22]. The fold-change calculation was based on the ratio of the mean of the normalized counts of permanent and primary teeth PDLs [mean (perm-PDLs) / mean(dec-PDLs)]. The final list of significant proteins was submitted to functional annotation tool of DAVID program (Database for Annotation, Visualization and Integrated Discovery), version 6.7, in order to identify the biological processes, cellular components and molecular functions (http://david.abcc.ncifcrf.gov/home.jsp) [23,24]. We only considered the enriched Gene Ontology (GO) terms (level 1) generated by modified Fisher Exact test followed by the Bonferroni test and *p* value threshold of < 0.05. In addition, gene and protein expression data in the PDL tissues were assessed by either the Student’s t-test with significance levels adjusted for 5%.

## Results

### 3.1 DecPDL and PermPDL cells produced mineral nodules *in vitro*

**Fig 1:**
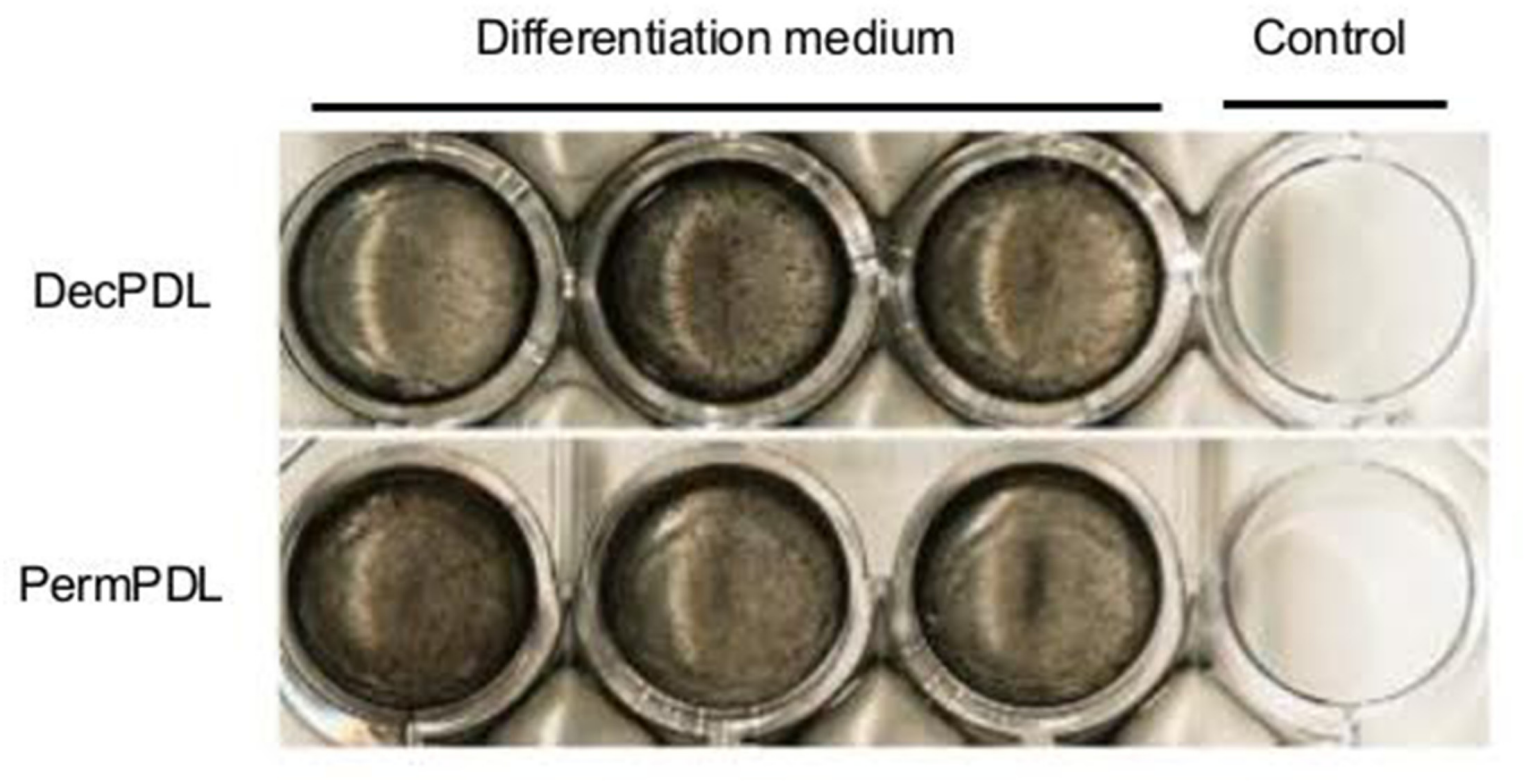
Mineralization *in vitro*. As expected, *Von Kossa* assay demonstrated that both, deciduous (DecPDL) and permanent (PermPDL) cells produced mineral nodules *in vitro* when cultured under osteogenic conditions.

Before performing the comparative constitutive secretome analysis of DecPDL and PermPDL cells, we wanted to functionally characterize these cells, and used the Von Kossa assay to demonstrate the potential of DecPDL and PermPDL cells to form mineral-like nodules *in vitro*. The results from the Von Kossa assay demonstrated that, indeed, all the DecPDL and PermPDL cell populations used in the present study were able to produce mineral-like nodule when maintained under osteogenic conditions (Fig 1), and therefore, confirmed the DecPDL and PermPDL cells with the expected phenotype.

### 3.2 Global proteome characterization of the secretome of PDL cells harvested from deciduous and permanent teeth

**Fig 2:**
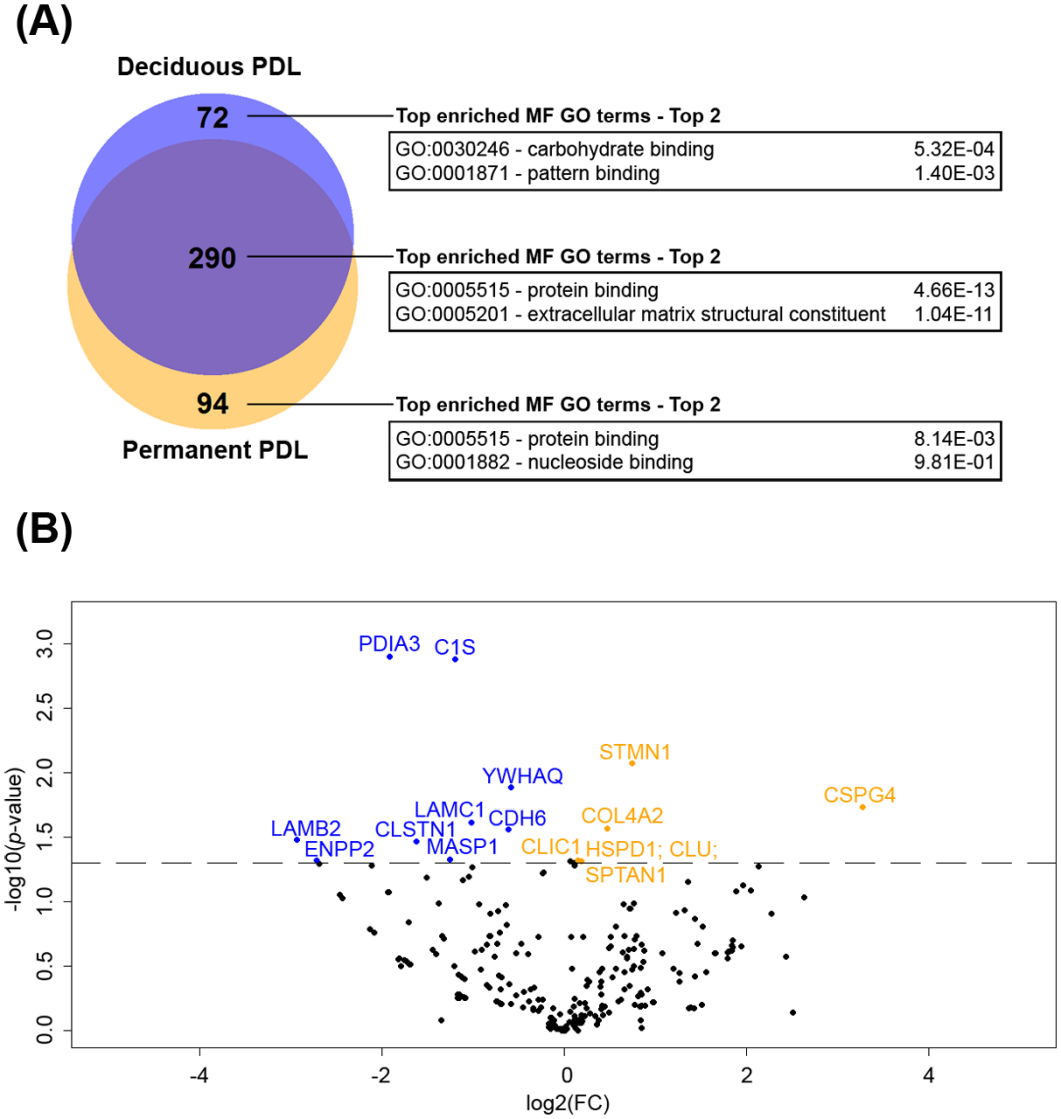
Summary of secretome analysis of the PDL cells harvested from deciduous and permanent teeth. **(A)** Venn diagram of total proteins and their top enriched molecular function (MF) GO terms. The MF GO terms were generated by DAVID software based on biological processes (GO_TERM_MF_2 database). **(B)** Volcano plot analysis of deciduous and permanent PDL cells secretome. Regulated proteins are painted in blue and orange, for deciduous and permanent teeth, respectively (*p*<0.05).

For a comparative analysis of proteins identified on the DecPDL and PermPDL cells, we performed a label-free quantitative proteomic analysis of samples of culture medium from three independent replicates of six healthy volunteers. In total, 456 total proteins were detected. Of the 456 proteins, 72 (15.79%) proteins were exclusively identified in culture medium from DecPDL cells, whereas 94 (20.61%) proteins were uniquely identified in PermPDL samples. A total of 290 proteins were identified in samples from both DecPDL and PermPDL cells. Of the 456 total proteins, 16 (3.5%) proteins were significantly differentially expressed (S1 table, Beta-binomial analysis, alpha = 5%). Among those 16 proteins, 9 (56.25%) proteins were more expressed in DecPDL cells [including LAMC1 (laminin subunit gamma 1) and LAMB2 (laminin subunit beta 2) (*p*<0.05)] and 7 (43.75%) proteins were more expressed in PermPDL samples [including CSPG4 (chondroitin sulfate proteoglycan 4) and COL4A2 (collagen alpha-2(IV) chain)] (*p* < 0.05) (Fig 2 and table 2).

**Table 2:** List of differentially expressed proteins in permanent and deciduous determined by beta-binomial statistical test (*p*-value < 0.05).

Protein Name	Protein Symbol	Permanent PDL mean (normalized spectral count)	Deciduous PDL mean (normalized spectral count)	p-value
Stathmin	STMN1	1.681	0	8.42E-03
Collagen alpha-2(IV) chain	COL4A2	1.393	0	2.68E-02
Isoform 2 of Spectrin alpha chain. non-erythrocytic 1	SPTAN1	1.112	0	4.73E-02
Isoform 2 of Clusterin	CLU	1.112	0	4.73E-02
60 kDa heat shock protein. mitochondrial	HSPD1	1.109	0	4.77E-02
Chloride intracellular channel protein 1	CLIC1	1.141	0	4.82E-02
Chondroitin sulfate proteoglycan 4	CSPG4	3.071	0.316	1.84E-02
Laminin subunit gamma-1	LAMC1	5.070	10.250	2.43E-02
Complement C1s subcomponent	C1S	8.990	20.607	1.31E-03
Isoform 2 of Mannan-binding lectin serine protease 1	MASP1	3.646	8.707	4.72E-02
Isoform 2 of Calsyntenin-1	CLSTN1	1.422	4.360	3.38E-02
Isoform 2 of Ectonucleotide pyrophosphatase/phosphodiesterase family member 2	ENPP2	0.286	1.877	4.75E-02
Laminin subunit beta-2	LAMB2	0.283	2.161	3.27E-02
Isoform 2 of Cadherin-6	CDH6	0	1.526	2.72E-02
14-3-3 protein theta	YWHAQ	0	1.501	1.30E-02
Protein disulfide-isomerase A3	PDIA3	0	3.755	1.26E-03

### 3.3 Biological characterization of identified proteins

**Fig 3:**
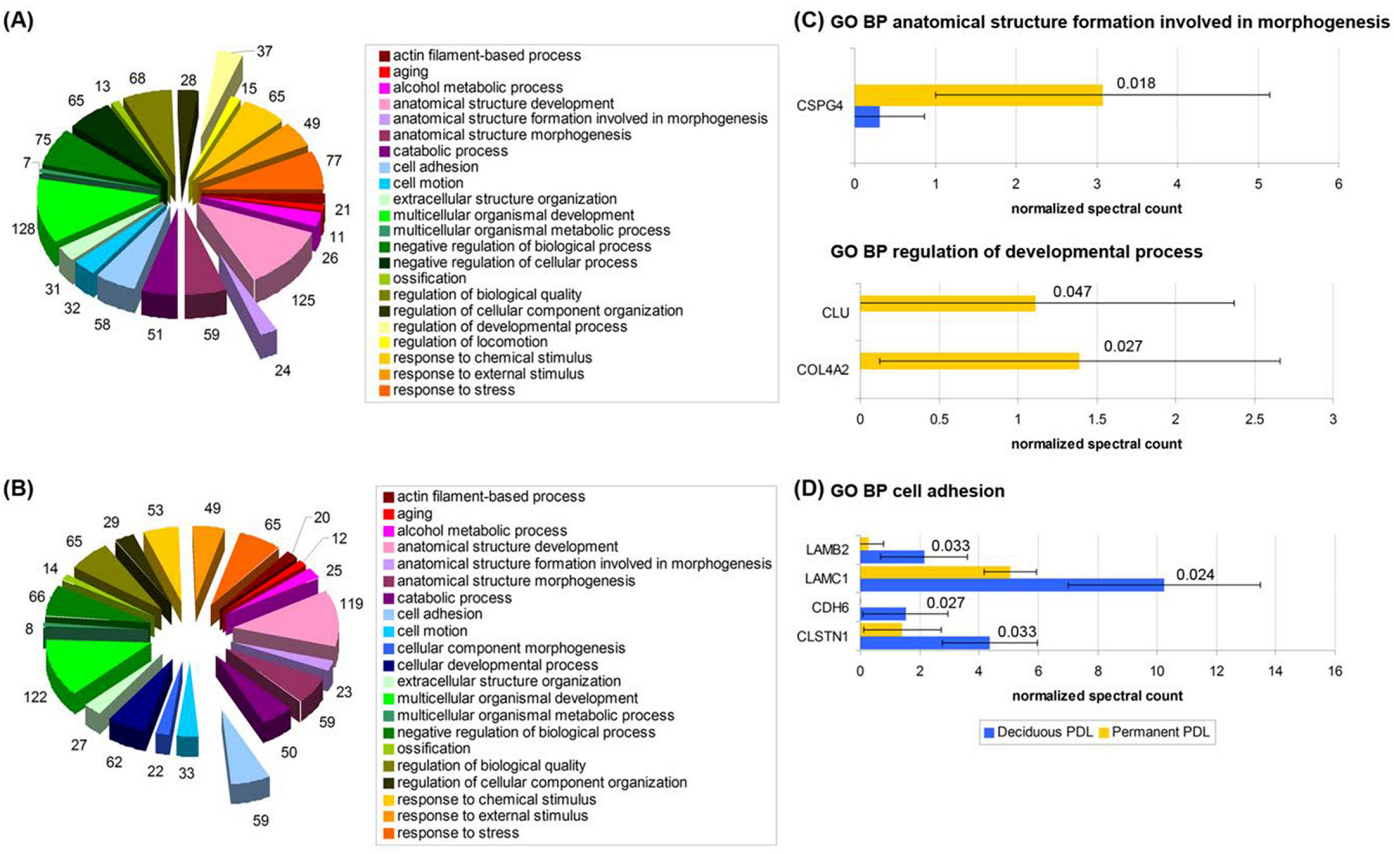
Gene Ontology (GO) enrichment analysis of the secretome of deciduous and permanent PDL cells. GO analysis was generated by the DAVID software based on biological processes (GO_TERM_BP_2 database). **(A)** and **(B)** GO biological process (BP) analysis from permanent and deciduous PDL cells secretome, respectively. **(C)** Barplot of normalized spectral count of differentially expressed proteins in permanent PDL cells with GO BP anatomical structure formation involved in morphogenesis and GO BP regulation of developmental process (mean ± standard deviation and *p*-value determined by beta-binomial statistical test). **(D)** Barplot of normalized spectral count of differentially expressed proteins of deciduous PDLs involved with GO BP cell adhesion (mean ± standard deviation and *p*-value determined by beta-binomial statistical test).

Next, in order to understand the functional significance of the identified proteins, it was performed the GO enrichment analysis using the “Database for annotation, visualization, and integrated discovery” (DAVID) based on GO_TERM_BP2 (for biological process, level 2) database with p value < 0.05 and Bonferroni correction. Because GO enrichment analysis for cellular component and molecular function in PermPDL and DecPDL revealed a highly similar proteomic profile (data not shown), we focused our analysis on the biological process. Fig 3 illustrates the GO analyses. Biological processes enriched in the secretome of PermPDL included anatomical structure development [125 proteins, including CLU (Isoform 2 of Clusterin), CSPG4 and STMN1 (Stathmin)], negative biological process [75 proteins, including CO4A2 and SPTAN1 (Isoform 2 of Spectrin alpha chain, non-erythrocytic 1], anatomical structure formation involved in morphogenesis (24 proteins, including CSPG4), regulation of developmental process (37 proteins, including CLU and COL4A2), and regulation of cellular component organization (28 proteins, including CLU and SPTAN1) (Fig 3A and 3C). On the other hand, significantly enriched biological processes in DecPDL included cell adhesion [59 proteins, including LAMB2, LAMC1, CDH6 (Isiform 2 of Cadherin 6) and CLSTN1 (Isoform 2 of Calsyntenin 1)], cell motion [33 proteins, including LAMC1 and ENPP2 (Isoform 2 of Ectonucleotide pyrophosphatase/phosphodiesterase family member 2)], cellular component morphogenesis (22 proteins, including LAMB2 and LAMC1), and regulation of biological quality [65 proteins, including LAMB2 and PDIA3 (Protein disulfide-isomerase A3)] (Fig 3B and 3D). In summary, GO enrichment data analysis demonstrated a highly similar constitutive proteomic profile of DecPDL and PermPDL cells, with the most striking differences observed for negative biological process, anatomical structure development and cell adhesion GO enriched groups.

### 3.4 Expression of LAMC1 (laminin subunit gamma 1) was confirmed to be higher in DecPDL versus PermPDL tissues by Western blotting analysis

**Fig 4:**
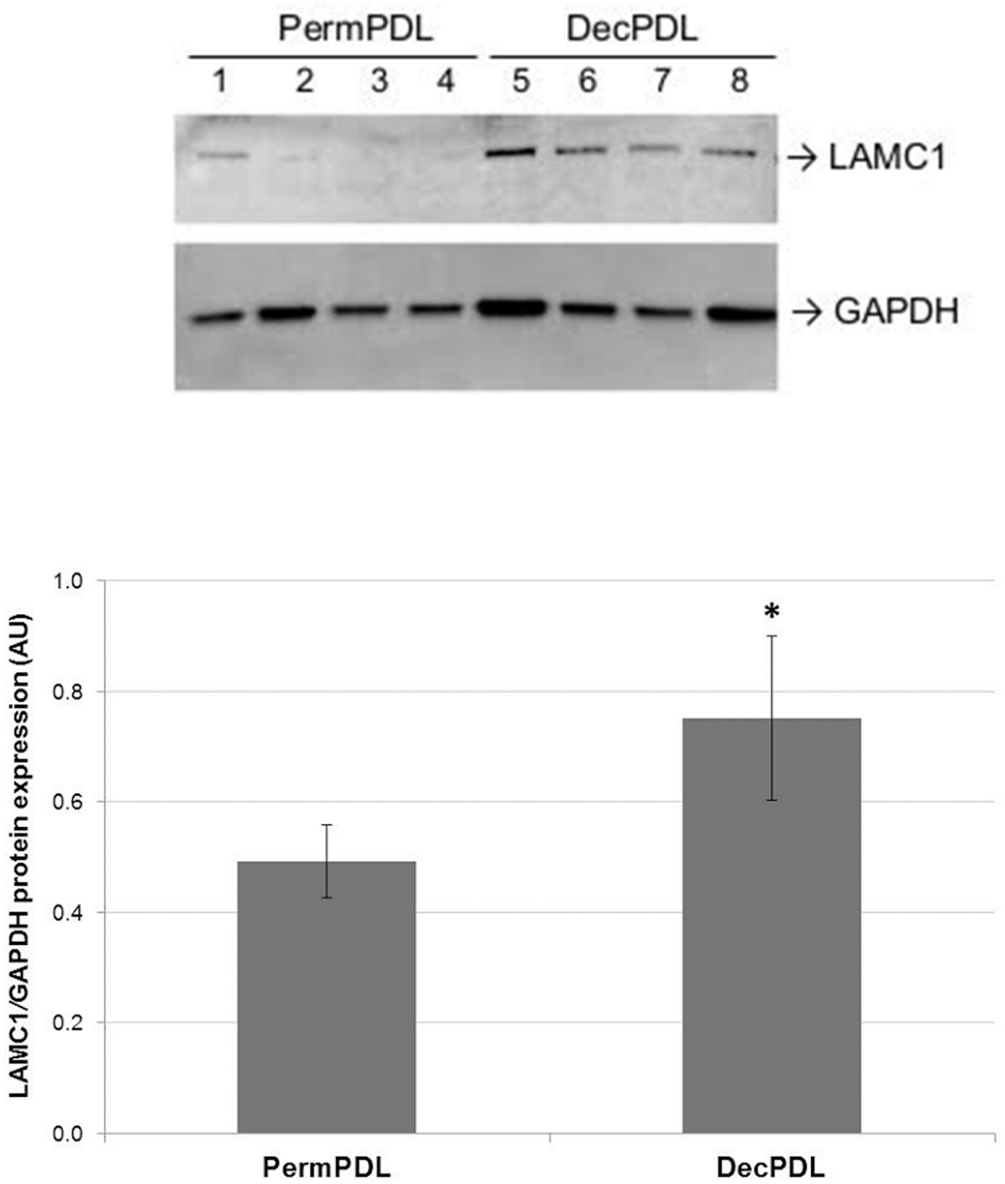
LAMC1 (laminin subunit gamma 1) protein levels in fresh PDL tissues. Western blotting analysis (four representative samples for each group) of the protein levels of LAMC1 in fresh PDL tissues harvested from deciduous (DecPDL) and permanent (PermPDL) teeth. Bar graph represents average and standard deviation of six samples per group. * indicates statistical difference by Student’s t-test with α = 5%.

In the secretome analysis, we found that LAMC1 was significantly increased in DecPDL cell culture medium compared to PermPDL cells (p<0.05), and these findings prompt us to validate our results in fresh PDL tissues obtained from deciduous and permanent teeth. Western blot analysis clearly confirmed that LAMC1 levels were increased (≅37.5%) in protein extracts obtained from fresh PDL tissues from deciduous compared to permanent teeth (p<0.05—Fig 4).

### 3.5 Laminin chains are differentially expressed in the PDL tissues of deciduous and permanent teeth

**Fig 5:**
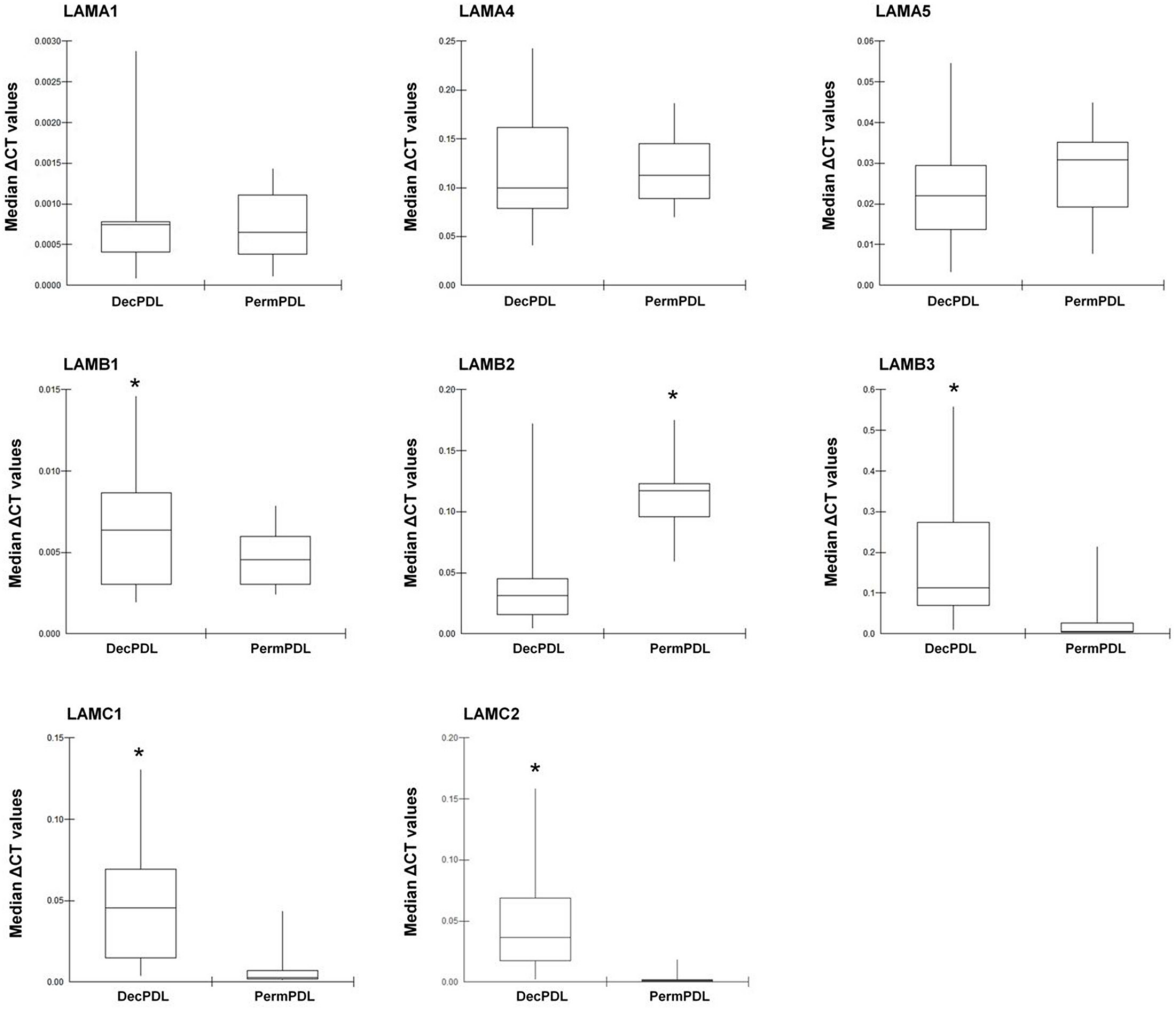
Gene expression in fresh PDL tissues for laminin chains. qPCR analysis of mRNA levels for laminin (LAM) chains in fresh PDL tissues harvested from deciduous (DecPDL) and permanent (PermPDL) PDL tissues reveals that deciduous teeth presented higher levels of *LAMB1* (laminin subunit beta 1), *LAMB3* (laminin subunit beta 3), *LAMC1* (laminin subunit gamma 1), and *LAMC2* (laminin subunit gamma 2), and lower mRNA levels of *LAMB2* (laminin subunit beta 2). * indicates statistical difference by Student’s t-test with α = 5%.

Based on the secretome and Western blotting findings, we hypothesized that laminin chains are differentially expressed in PDL tissues from deciduous and permanent teeth. To test our hypothesis, we isolated total RNA from fresh PDL tissues harvested from deciduous and permanent teeth and comparatively assessed the expression of the following genes by qPCR: *LAMA1*, *LAMA4*, *LAMA5*, *LAMB1*, *LAMB2*, *LAMB3*, *LAMC1*, and *LAMC2*. Data analysis demonstrated that, indeed, laminin chains are differentially expressed in the fresh PDL tissues of deciduous and permanent teeth (Fig 5). *LAMB1*, *LAMB3*, *LAMC1*, and *LAMC2* mRNA levels were significantly higher in the fresh PDL tissues obtained from deciduous teeth, whereas *LAMB2* transcripts levels was higher tissues harvested from permanent teeth (p<0.05).

### 3.6 Morphological analysis reveals that PDL tissues from deciduous and permanent teeth may be morphologically distinct

**Fig 6:**
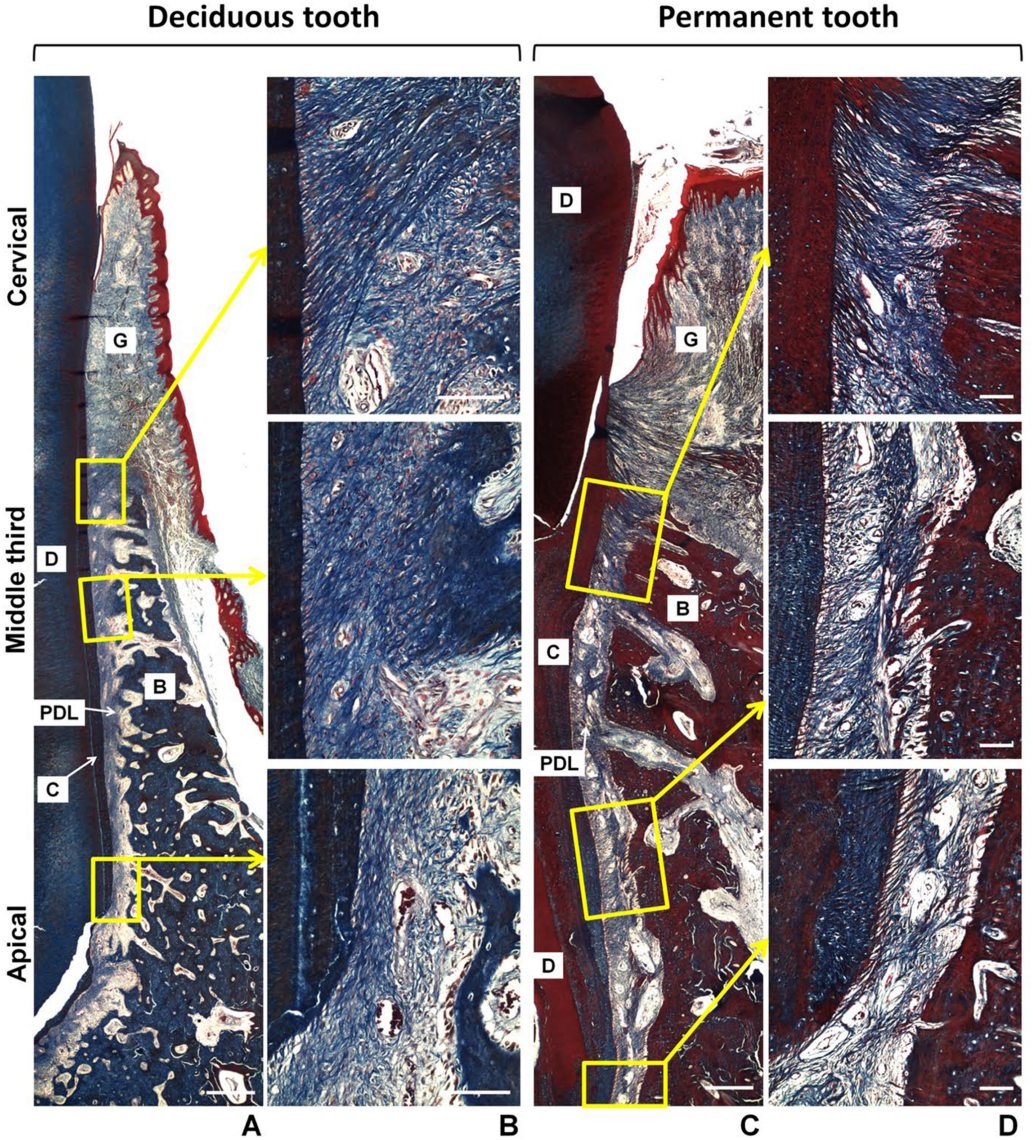
Histological illustration of deciduous and permanent teeth from mini pigs stained by Mallory’s trichrome. The general structure of the periodontium and collagen organization is shown in a panoramic view of the lingual face of deciduous **(A)** and permanent **(C)** teeth. The areas delimited by yellow squares are shown in columns **B** and **D** in higher magnification of the cervical, middle third and apical region of deciduous and permanent teeth, respectively. A and C, bars = 500 μm; B and D, bars = 100 μm. **G**—gingiva; **D**—dentin; **B**—bone; **C**—cementum; **PDL**—periodontal ligament.

**Fig 7:**
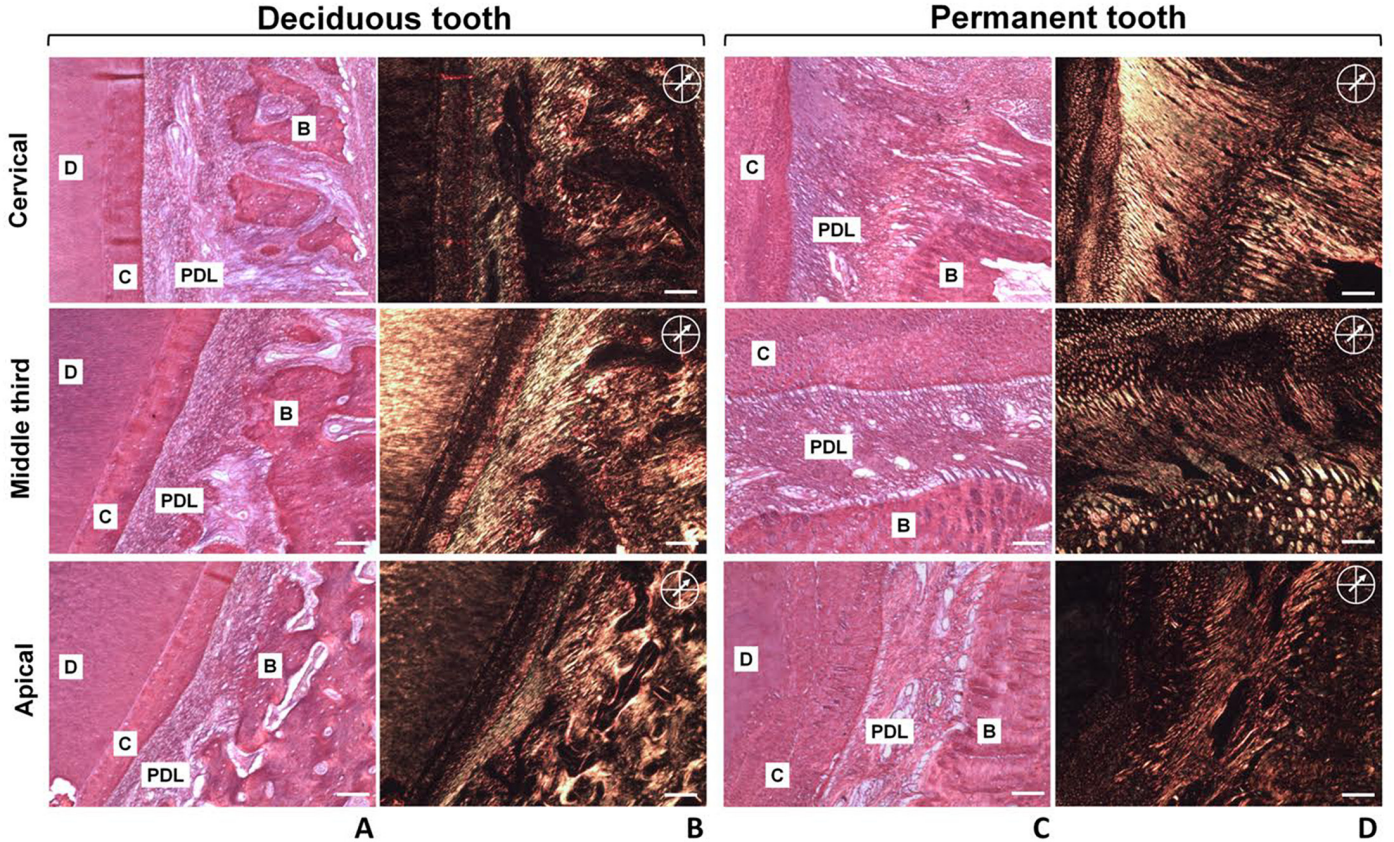
Polarizing and bright-field images from picrosirius red stained sections from deciduous and permanent teeth. Bright field images of the cervical, middle third and apical regions of deciduous **(A)** and permanent **(C)** teeth, and their respective birefringence in **B** and **D**. The arrow at 45° with the polarizer and analyzer indicates position of maximum birefringence. Magnification = 10x. Bars = 100 μm. **D**—dentin; **B**—bone; **C**—cementum; **PDL**—periodontal ligament.

The fact that we found a distinct expression pattern of laminin chains in the PDL tissues/cells from deciduous and permanent teeth, prompt us to assess potential morphological dissimilarities between deciduous and permanent PDL tissues. Thus, we performed qualitative histological analysis of the periodontal tissues of deciduous and permanent teeth harvested from minipigs. Mallory’s trichrome staining was used to assess the collagen fibrillar organization and structure of the periodontal tissues (Fig 6), and in general, histological analysis revealed thicker gingivae, alveolar bone and periodontal ligament area for permanent versus deciduous teeth. Several incremental lines in dental cementum, a larger number of cementocytes, larger alveolar bone trabecular spaces with large blood vessels entering the PDL tissue were found for permanent teeth. In contrast, histological analysis of the PDL tissues in deciduous teeth demonstrated that blood vessels appeared to be localized in a larger number adjacent to the alveolar bone and that collagen fibers are highly concentrated in areas adjacent to dental cementum. Picrosirius-red-stained sections viewed under polarized light (Fig 7) reveal a higher collagen fibers birefringence pattern in the PDL tissues of permanent teeth, suggesting a higher organizational pattern than deciduous teeth. In addition, in permanent teeth, the PDL tissues presented with thicker bundles of collagen fibers at the attaching the alveolar bone to the dental cementum in the cervical region (alveolar crest fibers), and larger bundles of Sharpey’s fibers inserting the alveolar bone and dental cementum in the mid third of the PDL tissues. No significant morphological/organizational differences were found with respect to the horizontal, oblique and apical fibers of the PDL tissues.

## Discussion

The periodontium can be considered as a system composed of hard tissues, such as dental cementum and alveolar bone; and vascularized soft tissues, which include the periodontal ligament. The periodontal ligament is a fibrous, complex, soft connective tissue machinery that connects two distinct hard tissues, the alveolar bone and the tooth root surface. It has been proposed to play a crucial role in allowing the teeth to withstand masticatory forces, to act as a sensory organ and to serve as a cell reservoir for tissue homeostasis and regeneration [1]. Given the functional differences between DecPDL and PermPDL tissues, it is reasonable to assume that these two tissues will display a distinct protein expression pattern, which will potentially account for their specific physiological functions. Although, differential expression of some molecules has been suggested [11–15,18], these findings are not sufficient to stand-alone and explain the physiological distinctions between the normal-functioning DecPDL and PermPDL, and therefore, additional studies are needed. Here, using a label-free quantitative proteome analysis we compared the abundance of secreted proteins present in primary cell culture of DecPDL and PermPDL. Our analyses by label-free liquid chromatography-mass spectrometry were paralleled by qPCR and Western blotting analysis directed to the expression of laminin chains in DecPDL and PermPDL fresh tissues. Our studies revealed that among the commonly expressed proteins, only 3.5% (16 proteins) were differentially expressed/secreted by DecPDL and PermPDL, and clearly indicated that while secretome differences indeed exist between these cells, they presented a highly similar pattern. In the current investigation, data analysis further demonstrated that 72 (15.79%) and 94 (20.61%) of the secreted proteins by primary PDL cells were exclusively expressed in DecPDL and PermPDL, respectively; with the top two enriched molecular function proteins enriched in carbohydrate and pattern binding, and protein and nucleoside binding in the DecPDL and PermPDL secretomes (Fig 2).

Clinically, because the most evident distinction between deciduous and permanent dentitions is the physiological loss of the deciduous tooth due to root resorption, we turned our attention to a potential differential expression of tooth root resorbing-associated agents and matrix-degrading factors in the secretome of DecPDL and PermPDL. Importantly, in the current investigation, secretome analysis did not detect differences between DecPDL and PermPDL regarding the expression of either tooth root-resorbing factors or matrix-degrading factors, such as RANKL, OPG and MMPs (matrix metalloproteinases). In contrast to previous reports [11–14], our findings of no difference in those factors between DecPDL and PermPDL is probably due to the fact that we did not include root-resorbing PDL areas for either cell isolation or fresh tissue collection, which seems to be in line with the findings by Fukushima et al. (2003) and Song et al. (2013)[15,18].

Because of the highly similar secretome profile evidenced by GO enrichment analysis for cellular component and molecular function, we decided to concentrate our GO enriched analysis on biological processes for the differentially expressed proteins. In general, among the differentially regulated factors, GO enrichment analysis by biological processes, revealed that highly expressed proteins in primary PermPDL cells were associated with apoptosis (SPTAN1 and CLU) and nervous system (STMN1), whereas highly expressed molecules in the DecPDL group were associated with cell adhesion (LAMC1, LAMB2, CDH6 and CLSTN1) and immune response (C1S and MASP1). This approach revealed that about 25% of the 16 differentially expressed proteins were higher in DecPDL and were involved with cell adhesion. These results prompt us to expand our studies to fresh PDL tissues harvested from deciduous and permanent teeth to validate our findings. We found that LAMC1 protein levels and *LAMB1*, *LAMB3*, *LAMC1*, and *LAMC2* mRNA levels are indeed higher, whereas *LAMB2* transcripts levels was lower in fresh DecPDL tissues compared to PermPDL. Song et al. (2013), using a microarray approach, examined and compared gene expression profile of fresh PDL tissues harvested from non-resorbing areas of deciduous and permanent teeth, and similarly to our findings, reported increased transcripts levels of genes encoding LAMC2 and LAMB3 [18].

Laminins are large molecular weight glycoproteins constituted by the assembly of disulfide-linked polypeptides (α, β and γ chains). The human genome encodes 11 distinct laminin chains, with both common and specific functions. One of the most important and common functions of laminins is to interact with proteins anchored in the plasma membranes of cells relaying biochemical and mechanical signals between extracellular and intracellular molecular networks, and therefore regulating multiple cellular activities and signaling pathways. Some not yet well-defined parts of laminins are thought to interact with small molecules such as cytokines and growth factors, and that is suggested to be an important function for sequestration and storage of these small molecules and for controlling their distribution, activation and presentation to cells. Furthermore, laminins and related extracellular matrix components have essential roles in wound healing and integrin-mediated interactions with laminins are important for several cellular activities [25,26]. Analyses of the various laminin deficient mouse models and laminin-associated human disorders have helped us to improve our understanding of the function of the individual laminins. This is exemplified by the phenotypes associated with spontaneous, targeted and induced mutations in mouse in the *Lama2* gene, and by human disorders associated with mutations in the *LAMA3*, *LAMB3* and *LAMC2* genes [27,28].

Laminin expression has been previously documented in the dental tissues, including the PDL tissues by immunolocalization assays [29] and PDL cells *in vitro* by Western blotting and RT-PCR [30]. Because odontoclast adhesion, activation and subsequent root resorption are thought to be associated with the temporospatial expression and maturation of various extracellular matrix proteins [31–33], one can speculate that a distinct and differential expression pattern of laminins on deciduous versus permanent tooth roots may serve as pattern to signal to a selective resorption of deciduous roots during physiologic tooth eruption. However, to the best of our knowledge, no tooth phenotype has been described in the case that laminins are not functional in the PDL region. Our morphological findings suggest that a distinct expression pattern of laminin chains in the PDL region may account for potential distinctions in the organization and physiology of the periodontium between deciduous and permanent teeth. Together, these findings provide evidence to support an involvement of laminins, and laminin-dependent pathways, in the control of physiological differences between the PDL tissues in deciduous and permanent teeth. However, additional mechanistic-oriented studies are necessary in order to determine the potential roles of laminins on the control of biological events associated with either deciduous or permanent tooth root physiology.

## Supporting Information

S1 Table Spectrum count report and statisctical results of all identified proteins per sample (n = 3 DecPDL and n = 3 PermPDL). Fold change and p-values defined by the beta-binomial test are shown.
(XLSX)
